# On the Evolution of Specificity in Members of the Yeast Amino Acid Transporter Family as Parts of Specific Metabolic Pathways

**DOI:** 10.3390/ijms19051398

**Published:** 2018-05-08

**Authors:** Christos Gournas, Alexandros Athanasopoulos, Vicky Sophianopoulou

**Affiliations:** Microbial Molecular Genetics Laboratory, Institute of Biosciences and Applications (IBE), National Centre for Scientific Research “Demokritos” (NCSRD), Patr. Grigoriou E & 27 Neapoleos St., 15341 Agia Paraskevi, Greece; alexandr@bio.demokritos.gr

**Keywords:** YAT, APC, amino acids, l-proline, *prn* cluster, Area, CreA, transcriptional regulation

## Abstract

In the recent years, molecular modeling and substrate docking, coupled with biochemical and genetic analyses have identified the substrate-binding residues of several amino acid transporters of the yeast amino acid transporter (YAT) family. These consist of (a) residues conserved across YATs that interact with the invariable part of amino acid substrates and (b) variable residues that interact with the side chain of the amino acid substrate and thus define specificity. Secondary structure sequence alignments showed that the positions of these residues are conserved across YATs and could thus be used to predict the specificity of YATs. Here, we discuss the potential of combining molecular modeling and structural alignments with intra-species phylogenetic comparisons of transporters, in order to predict the function of uncharacterized members of the family. We additionally define some orphan branches which include transporters with potentially novel, and to be characterized specificities. In addition, we discuss the particular case of the highly specific l-proline transporter, PrnB, of *Aspergillus nidulans*, whose gene is part of a cluster of genes required for the utilization of proline as a carbon and/or nitrogen source. This clustering correlates with transcriptional regulation of these genes, potentially leading to the efficient coordination of the uptake of externally provided l-Pro via PrnB and its enzymatic degradation in the cell.

## 1. Introduction

Amino acids are the building blocks of proteins and most fungi possess the ability to utilize them as nitrogen and/or carbon sources. To do so, they possess a wide repertoire of transporters for the uptake of amino acids from the extracellular medium and the cognate catabolic enzymes. For the last 50 years, work on model ascomycetes, mainly *Aspergillus nidulans* and *Saccharomyces cerevisiae* has led to the genetic and biochemical characterization of a plethora of general and specialized fungal amino acid transport systems (fAATs). Three eras can be distinguished in the discovery of transporters: (1) the classical genetics era, where pioneering work, based on the use of toxic substrate analogs, has led to the isolation of the first mutations in transporter genes. Paradigmatic examples are amino acid and purine transporters of *A. nidulans* [1,2,3], the Arg (Can1) and Lys (Lyp1) specific permeases of yeast, as well as the general amino acid permease, Gap1 [4,5,6] (2) the reverse genetics era, where the first sequences of several transporter genes have been cloned, including Can1, Gap1, the histidine (Hip1) and the proline (Put4) permeases of *S. cerevisiae* [7,8,9,10], as well as the proline-specific transporter, PrnB, and the γ-amino-*n*-butyrate transporter, GabA of *A. nidulans* [11,12] (3) the genomics era, where the whole genome sequences of several species, among the first being those of *S. cerevisiae* and *A. nidulans* [13,14], provided the amino acid sequences of a plethora of non-characterized transporter proteins. During this era, with the availability of the exponential expansion of genome sequences, transporter protein families have been defined (see below) by extensive phylogenetic analyses [15]. At the same time, the first crystal structures of membrane proteins (see below), in combination with 3D modeling and extensive site-directed mutagenesis, boosted the study of transporter structure-function relationships. These approaches have led to the characterization of specificity determinants in model fAATs, such as Can1 and PrnB [16,17]. Recently, comparative genomics of several closely related *Aspergillus species* has allowed the establishment of detailed phylogenetic relationships of fAATs [18]. A major challenge for the future is the functional characterization of the increasing pools of orphan fAAT sequences. This work, besides summarizing the existing knowledge on fAATs, also discusses the potential for combining comparative genomics with detailed structure-function studies of model transporters for the prediction of the specificity of non-characterized permeases.

Fungal AATs have been identified not only at the plasma membrane but also in subcellular organelles, namely the vacuolar and mitochondrial membranes, as well as intracellular vesicles (for review see [19]). fAATs belong to different protein families, several of which are phylogenetically related and grouped into two major superfamilies, the Aminoacid-Polyamine-organoCation (APC) superfamily (Transporter Classification database identifier, TC: 2.A.3) and the Major Facilitator Superfamily (MFS, TC: 2.A.1) [20]. The vast majority of these characterized plasma membrane-localized fAATs belong to the APC superfamily [19]. Within the APC, three major families are identified: The Yeast Amino acid Transporter (YAT) family (TC: 2.A.3.10) [21], the l-type Amino acid Transporter family (TC: 2.A.3.8) and the Amino acid/Choline Transporter family (2.A.3.4) [22,23].

## 2. The Yeast Amino Acid Transporter (YAT)-Family of Fungal Amino Acid Transporters

Most of the fAATs lie within the YAT family. This is also reflected in the number of YATs with known functions, as well as the extensive knowledge available regarding their regulation of expression (analyzed below) and structure-function relationships [19].

For all the members of the APC superfamily, YATs adopt the common fold of two intertwined repeats consisting from 5 TransMembrane (TM) segments each (also called the LeuT or 5 + 5 fold). In this fold, the two repeats (TMs 1–5 and 6–10 in YATs) show an inverted pseudo-symmetry. Two unwound segments within the first TM from each repeat (thus TM1 and TM6 in YATs) form the main substrate-binding pocket. A varying number of additional TMs also exist in several APC subfamilies. YATs possess two additional TMs (TM11 and TM12), whose roles remain unclear. The high conservation of this fold becomes apparent when the crystal structures of the first bacterial members become available, namely the amino acid transporter LeuT of the Neurotransmitter Sodium Symporter (NSS) family (TC: 2.A.22), the benzylhydantoin transporter Mhp1 of Nucleobase Cation Symporter (NCS1) family 1 (TC: 2.A.39), the galactose transporter vSGLT of the Solute Sodium Symporter (SSS) family (TC: 2.A.21), and the glycine-betaine permease BetP of the Betaine Choline Carnitine transporter (BCCT) family (TC: 2.A.15) [24,25,26,27]. Subsequently, it was predicted that using hydropathy profile alignments that the APC superfamily would also adopt this fold [28]. Based on this notion, the first model of a YAT protein, PrnB, was built using 3D modeling [29], providing the first mutational and kinetic evidence that the substrate-binding pocket of YATs is formed by residues in TM1 and TM6. More evidence on the fold of APC members came with the crystallization of three bacterial APC members, the AdiC arginine/agmatine antiporters (belonging to the Basic Amino Acid/Polyamine Antiporter Family, TC: 2.A.3.2), a broad-specificity proton-coupled amino acid transporter ApcT (member of the Archaeal/Bacterial Transporter family, TC: 2.A.3.6), and the Glutamate:GABA antiporter GadC (member of the Glutamate:GABA Antiporter Family, TC: 2.A.3.7) [30,31,32,33,34]. All the above APC transporters, including LeuT, were crystalized in different conformations of the transport cycle, providing a series of snapshots of the transport cycle, and significantly enriching the previous view of the alternating access model for the function of transporters [35,36,37,38,39]. More specifically, at least four conformations of APCs existence were confirmed by the existing crystal structures. These conformations are described as either outward-facing or inward-facing, depending on the side of the membrane that the substrate binding site is oriented to. Additionally, the crystal structures have indicated the existence of gates in APCs, controlling access to the substrate binding site. When a gate is open, the substrate binding pocket has access to the solvent, while closure of the gate results in an occluded conformation. A full transport cycle begins with the binding of the substrate in an outward-facing open conformation. This binding causes the closure of the outer gate to promote an inward-facing occluded conformation. Subsequently, major conformational rearrangements of several TMs make the transporter adopt an inward-facing occluded conformation. The opening of the inner gate results in an inward-facing open conformation, eventually resulting in the release of the substrate. Finally, the shift of the transporter back to an outward-facing open conformation completes the transport cycle, and the protein is ready to perform the next cycle [35,36].

### 2.1. Structural Modeling of YATs

**Figure 1:**
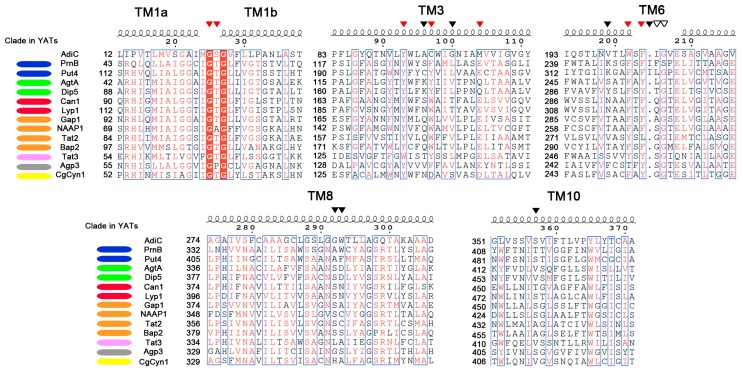
The important residues for substrate binding and determination of the specificity in the YAT family. A multiple alignment of the TMs1, 3, 6, 8 and 10 sequences from selected YAT members—for most of which structure-function studies have been performed. Residues in red font are highly conserved, while residues in red filled columns are absolutely conserved in the sequences of the selected YATs. Blue frames define residues that are conserved at least 70% of the sequences. Red triangles indicate the positions of residues that are highly conserved in YATs and are predicted to interact with the backbone of the amino acid substrates. Black triangles indicate specificity-determining residues that are poorly conserved in YATs and predicted to interact with the side chain of the amino acid substrates. Empty triangles indicate residues that indirectly affect the specificity of YATs. Color-coding next to the names of YATs shows which clade of the phylogenetic tree of Figure 2 the transporters belong to. The annotation of the alignment was done with ESPript 3.0 [45].

The availability of the first crystal structures of APCs triggered the detailed study of the structure-function relationships of several YATs. Studies combining 3D modeling, coupled to substrate docking, mutational, and kinetic analyses have provided strong evidence about the recognition of the substrate and the determination of the specificity in the YAT family. 3D modeling of YATs has been mainly performed on the structure of AdiC (belonging to TC: 2.A.3.2), given the close phylogeny to the YAT family (TC: 2.A.3.10), the reasonable sequence similarity, and the availability of structures for AdiC trapped in different states [30,32,34]. Up to now, 8 YATs in total have been modeled (Table 1). Six of them are from *S. cerevisiae*, the general amino acid permease Gap1 [40], the arginine and lysine permeases (Can1 and Lyp1, respectively) [16], the aromatic amino acid transporter Tat2 [41], the branched chain amino acid permease Bap2 [42], the proline transporters from both yeast and *A. nidulans* (Put4 and PrnB, respectively) [17], as well as one YAT from *Candida glabrata*, the cystine transporter CgCyn1 [43]. The above studies have identified a limited number of amino acid residues interacting with the substrate. These residues are found in specific and conserved positions of TM1, TM3, TM6, TM8 and TM10 (Figure 1). A multiple structural alignment of these TMs reveal that these residues can be grouped into two categories: (1) residues that are highly conserved in YATs and are predicted to interact with the backbone of the amino acid substrates and (2) residues that show poor conservation and interact with the specific side chains of amino acid substrates.

### 2.2. Determination of Specificity in YATs 

Residues from two conserved motifs in the unwound segments of TM1 and TM6 have been found by substrate docking of Arg in Can1, Pro in PrnB and Phe in Gap1 to interact with the invariant part of amino acid substrates [16,17,19]. Importantly, The GTG motif in TM1 and the (F/Y)(S/A/T)(F/Y)xGxE in TM6 (Figure 1) correspond to the main residues of AdiC mediating the interaction with the amino acid backbone, i.e., the α-carboxyl and α-amino groups [34]. The docking results are well supported by extensive mutational analysis of these residues in PrnB, Gap1, Tat2 and Bap2 (for a summary see [19]), whose substitution severely affects the kinetic characteristics of the transporters or even completely abolishes transport.

At variance with the residues of AdiC that interact with the backbone of the substrate, the residues interacting with the side chain of Arg (Ala96, Cys97 and Met103 in TM3, Trp202 and Ile205 in TM6, Trp293 in TM8 and Ser357 in TM10, described in [34]) correspond to poorly conserved residues within the YAT family (Figure 1, reviewed in detail in [19]). It thus seemed reasonable to speculate that residues in these positions could determine the specificity of YATs. This was demonstrated for Can1 and PrnB [16,17]. Both studies were based on the existence of highly similar in sequence YATs, but with different specificities (Lyp1 for Can1 and Put4 for PrnB), for the identification of residues interacting with the side chain of the substrate. More specifically, Ghaddar et al. showed that substitutions S176N in TM3 and T456S in TM10 were together sufficient to convert Can1 into a high affinity and capacity Lys specific transporter [16]. The rational design for this conversion was based on substrate docking of Arg in Can1 and Lys in Lyp1. This approach revealed that residues in these two positions are the only differences in the substrate binding pockets of Can1 and Lyp1. Similarly, the highly selective for l-Proline PrnB could be relaxed by appropriate substitutions such as the protein could now recognize and/or transport several additional molecules (l-Ala, Gly, l-azetidine-2-carboxylic acid), which are substrates of its orthologue, Put4, without affecting the high affinity for l-Pro [17]. This conversion required five Put4-mimicking substitutions in the substrate binding pocket of PrnB (S130C in TM3, F252L and S253G in TM6, W351F in TM8 and T414S in TM10), the resulting quintuple mutant showing a specificity profile very similar to that of Put4. Impressively, two of the five residues identified corresponded to the two residues determining the specificity of Can1 (Figure 1, Ser130 of PrnB corresponds to Ser176 of Can1, Thr414 of PrnB corresponds to Thr456 of Can1). The above finding strongly indicates that residues found in a limited number of positions could determine the specificity of YATs. This raises an interesting perspective: Is it possible to predict the specificity of non-characterized YATs based on residues in these positions? This question is discussed below. It is important to note, however, that besides the substrate binding core, other regions in YATs are also known to affect specificity (reviewed in detail in [19]). These regions are the proximal, middle, and distal gates, as well as some loops connecting TMs. Mutants affecting the specificity of several YATs have been isolated in these regions by classical, non-directed mutagenesis. The most plausible explanation is that the residues in these positions indirectly affect substrate recognition, e.g., by controlling accesses to the substrate binding core from extracellular and cytoplasmic sides.

### 2.3. Modeling the Conformational Changes in YATs

It is worth mentioning that the existing evidence from the study of both PrnB and Can1, indicate an additional important role for the specificity-determining residues in TM3 and TM10: affecting the substrate-induced structural transitions of the transport cycle in a synergistic way. More specifically, the single Put4-mimicking substitutions in PrnB are either thermosensitive (S130C in TM3) or significantly affected by transport (T414S in TM10), as seen by both radiolabeled l-Pro uptake measurements and growth in Pro as the sole source of nitrogen. Importantly, these phenotypes are not observed for the fully functional double S130C/T414S [17]. Similarly, the single Can1(S176N) is non-functional and this phenotype is rescued by the introduction of T456S [16]. Importantly, modeling and docking studies showed that Can1(S176N) is non-functional, though it is still predicted to be able to bind Arg, due to steric hindrance by the large side chain of Asn176. This steric hindrance, most probably not allowing the shift to an inward-facing conformation, is relieved by the T456S substitution [44]. Indeed, this was confirmed by two cases where the conformation adopted by Can1 could be experimentally verified. We recently presented evidence that the conformation of the transporter is important for both the recognition of Can1 by the Art1 α-arrestin during substrate-induced endocytosis [44], and for the partitioning of Can1 in the starvation-protective Eisosome Membrane Compartments (EMCs) [46]. In brief, in the absence of a substrate, Can1 is preferentially localized in EMCs, and presumably more populated in an outward-facing conformation. Upon the addition of Arg, Can1, which should become more populated in an inward-facing conformation, homogeneously dissipates to the plasma membrane. In this state, Can1 exposes a binding site for Art1 (Art1BS) at the N-terminal tail of the transporter, triggering the ubiquitylation, endocytosis and degradation of the transporter. The Art1BS is not accessible to Art1 in the absence of a substrate, when the transporter should predominantly adopt an outward-facing conformation. A mutant of Can1 [Can1 (E184Q)], shown by molecular modeling to be blocked in an inward-facing conformation, was found to be accessible to Art1 and not showing any preferential localization to EMCs even in the absence of substrate. Importantly, S176N is epistatic to E184Q for both phenotypes, which strongly indicates that S176N can block Can1 in an outward-facing conformation. Consistently, via molecular modeling, we have found that the presence of a bulky Asn residue at position 176 introduces steric hindrance at the outward-facing-occluded conformation, suggesting that this mutant should be deficient for transitioning to an inward-facing state of the transport cycle.

## 3. Phylogenetic Analyses Combined with the Conservation of Residues in Specificity-Determining Positions as a Guide for the Specificity of Transporters

**Figure 2:**
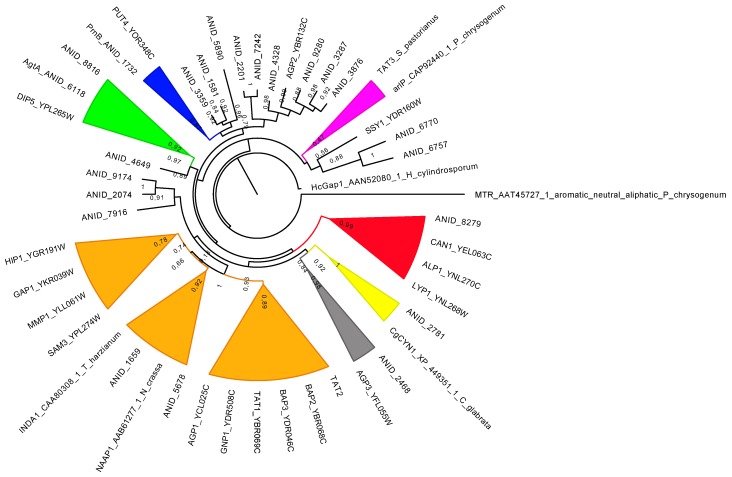
A phylogenetic tree of YATs. All YATs from *A. nidulans* and *S. cerevisiae* are included, together with other functionally characterized fungal transporters. The *S. cerevisiae* proteins TAT1, TAT2, TAT3, GAP1, HIP1, GNP1, AGP1, AGP2, AGP3, BAP2, BAP3, SAM3, MMP1, LYP1, ALP1, CAN1, DIP5, PUT4 and SSY1 were obtained from the Saccharomyces Genome Database (http://www.yeastgenome.org). The sequences of the YATs of *A. nidulans* were collected and manually corrected for errors occurring by predictions of exon splicing [18]. The previously detected pseudogenes [18], ANID_4604, ANID_8556, ANID_8659 and ANID_5024, are excluded from the tree. Other proteins included are two characterized broad specificity amino acid permeases from *Neurospora crassa* (NAAP1) [47] and *Trichoderma harzianum* (INDA1) [48], one Aromatic and one Aromatic and aliphatic amino acid transporter from *Penicillium crysogenum* (MTR, ArlP) [49,50], a general amino acid permease from *Hebeloma cylindrosporum* (HcGap1) [51] and a cystine-specific transporter from *Candida glabrata* (CgCyn1) [52]. Alignment was performed with MAFFT G-INS-I with default parameters [53], alignment curation with BMGE with default parameters [54]. A maximum likelihood tree was obtained with PhyML (http://www.atgc-montpellier.fr/phyml/) with automatic selection substitution model (LG) [55]. The tree was drawn with FigTree (FigTree v1.4.2. http://tree.bio.ed.ac.uk/software/figtree/). The tree shown is in a simplified cartoon form in which several branches are collapsed into triangles, only when related to a functionally characterized homologue. Color codes. Red: basic amino acid transporters; green: dicarboxylic amino acid transporters; yellow: putative cystine transporters; dark grey: putative branched amino acid transporters; dark purple: aromatic amino acid transporters; Dark blue: proline transporters; orange: transporters related to a functionally diverse group of structurally related *S. cerevisiæ* transporters. Within this latter group, there is a subclade of characterized general amino acid transporters from different species. A more comprehensive tree comprising the YATs of all available Aspergilli can be found at Additional file 23 of [18].

We have recently performed a phylogenetic analysis of all the YATs coming from *Aspergillus* species with known genomes [18]. A smaller tree is shown in Figure 2, containing all YATs of known function, as well as all the YATs of *A. nidulans* and *S. cerevisiae*. It is evident from both trees that in several cases, specificity arose prior to the divergence of the Saccharomycotina and the Pezizomycotina. For example, it is plausible that transporters in certain clades have defined specificities, based on the existence of at least one transporter with a known function. Clades containing transporters specialized in the uptake of basic (red), aromatic (purple) or dicarboxylic amino acids (green), cystine (yellow) and proline (blue) can be distinguished. In some cases (transporters specific for proline, aromatic and dicarboxylic amino acids, as well as broad specificity permeases), this is validated by the clustering of more than one characterized transporters from different species. Additionally, a clade of general amino acid permeases from different species (orange, containing Gap1) can be defined. Interestingly, this clade contains only 1–2 transporters from each *Aspergillus* species but 10 YATs from *S. cerevisiae*. Thus, there seems to be a great expansion of this branch in *S. cerevisiae*, which occurs after the separation of Saccharomycotina and Pezizomycotina. Most of them are broad specificity permeases, however some specialized transporters are also present (Hip1, Tat2, Sam3, Mmp1). This expansion, via convergent evolution, could potentially compensate for the lack of transporters from several other clades in *S. cerevisiae*.

From the above findings, it is evident that co-clustering in the same clade is not always enough to assume the specificity of a given YAT. However, a detailed phylogenetic analysis (Figure 2 and [18]), combined with the conservation of specificity-determining residues (Figure 1) are strong indications for the specificity of a YAT. For example, it is evident that the residues occupying the specificity-determining positions of Put4 and PrnB are very similar (Figure 1), while a few differences have been shown to be largely dependent on the small differences in specificity between the two transporters [17]. The same also applies to Can1 and Lyp1, the two characterized basic amino acid transporters [16], while, as previously discussed [18], two sub-clades exist in the Aspergilli: one with Can1-like residues in these positions and another with Lyp1-like residues. The findings above suggest the potential existence of Arg- and Lys-specific permeases in most of Aspergilli. It is also evident that transporters on the same clade sharing the same specificities possess very similar residues in the specificity-determining positions (Figure 1): for example AgtA and Dip5 [56], the two dicarboxylic amino acids transporters, or Gap1 and NAAP1 [9,47], the two general amino acid permeases from *S. cerevisiae* and *Neurospora crassa*. On the contrary, however as expected, transporters on different clades (Figure 2) and with different specificities show little to no similarity in the residues occupying the specificity-determining regions (Figure 1). Thus, the combined approach analyzed above could potentially be used to predict the specificities of non-characterized transporters, but also to unravel the fine differences in the specificities of orthologues. This requires the presence of a closely related transporter with a known function. However, the trees of YATs show the existence of several clades (Figure 2 and [18]) consisting exclusively of the orphan members. The residues occupying the specificity-determining positions of these orphan members show no obvious similarity with any characterized transporter (our unpublished observations). Thus, although the convergent evolution of protein function within the YAT family cannot be excluded, these orphans could possess entirely novel specificity profiles. For example, among these orphan transporters, there could be a permease responsible for the uptake of the proline analogue l-azetidine-2-carboxylic acid (AZC). AZC is a natural occurring plant-protectant that is, not only non-toxic for *A. nidulans*, but can also be used as a poor source of nitrogen (A. Biratsi, C. Gournas, A. Athanasopoulos, and V. Sophianopoulou, manuscript in preparation). Currently, no AZC transporter has been characterized in *A. nidulans*, while the obvious candidates due to the similarity of substrates, like the PrnB proline transporter, or the GabA GABA permease do not seem to significantly contribute to the transport of AZC [17].

Finally, it is worth mentioning that not all Aspergilli have clear orthologues of PrnB and Put4. The detailed phylogenetic tree shows that Put4 defines a clade consisting of only seven proteins from seven different *Aspergillus* species [18]. Interestingly, in all these species, the gene coding for these transporters is physically clustered with the genes coding for enzymes and transcriptional regulators required for the catabolism of Pro (see below). The physiological significance of this is discussed below. To our knowledge, this is the only case of such clustering, but since most YATs of *A. nidulans* remain uncharacterized, additional cases cannot be excluded. In fact, such clustering could be most informative for the specificity of the transporter, and could potentially be used for the prediction of the function of orphan YAT members. 

## 4. Regulation of YAT Expression—The *prn* Cluster: Efficient Coordination of Proline Uptake and Assimilation

Thirty years of work have revealed extensive details on the regulation of model YATs by carbon and nitrogen availability, both at the transcriptional (analyzed in detail for PrnB below) but also at the post-translational level. It has been shown for many YATs that their abundance at the plasma membrane is tightly coordinated at the protein level (reviewed in [19]). Gap1, Can1 and PrnB are prominent examples of this. For example, PrnB and AgtA have been shown to undergo endocytosis from the plasma membrane in response to the presence of the preferable nitrogen source ammonium (Am) [56,57], a phenomenon also described for purine transporters of *A. nidulans* [58]. The same also applies to Gap1, the paradigmatic permease for this regulation / process in *S. cerevisiae*. In brief, Gap1 Am-induced downregulation is mediated by its ubiquitylation by the Rsp5 Ubiquitin (Ub) ligase [59]. Recognition of Gap1 by Rsp5, as for most transporters in *S. cerevisiae* and *A. nidulans* [60,61], requires adaptor proteins of the α-arrestin family, namely the Bul1/2 for Gap1 [62,63,64]. The interaction of Bul1/2 with Gap1 is regulated by the availability of internal amino acids via the Target of Rapamycin Complex 1 (TORC1)-Npr1 signaling cascade [64]. Irrespective of the presence of Am, several transporters are known to undergo Rsp5-mediated ubiquitin-dependent endocytosis in the presence of high levels of their substrates [19,40,58,65,66]. For Can1 in particular, this has recently been shown to require a substrate-induced shift of the transporter to an inward-facing conformation of the transport cycle, exposing a binding sequence for the Art1 α-arrestin at the N-terminal tail of the transporter [44]. This endocytosis is crucial for optimal utilization of externally provided nutrients and avoidance of toxicity due to substrate over-accumulation [44]. Interestingly, it has been suggested on theoretical grounds that a combination of transporter transcriptional up-regulation and post-translational down-regulation is required for achieving “perfect homeostasis”, a condition where the intracellular concentration of a given substance is independent of all extracellular concentrations of this substance [67]. This combined mode of regulation has been experimentally described in detail for UapA, the uric acid transporter of *A. nidulans* [58]. It is noteworthy that a new level of regulation of transporter protein abundance has been very recently described: protection from endocytosis by partitioning in specific domains of the plasma membrane, the EMCs. Transporters in EMCs are protected from bulk endocytosis occurring in parallels to autophagy during starvation and stationary phase. This protection preserves a sub-population of transporter molecules from downregulation which allows cells to efficiently re-initiate growth once the nutrients become available [46].

**Figure 3:**
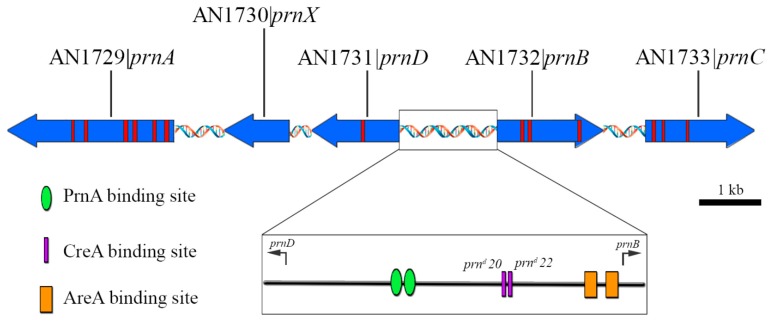
Schematic representation of the *prn* gene cluster organization in chromosome VII of *A. nidulans*. *prnB* (AN1732) encodes the specific proline transporter, prnD (AN1731) codes for proline oxidase, *prnC* (AN1733) for Δ1-pyrroline-5-carboxylate dehydrogenase, *prnA* (AN1729) for the pathway specific transcriptional activator and *prnX* (AN1730) is a proline-inducible gene of unknown function (see text). Genes are presented in scale with their standard names followed by their systematic names in the AspGD bank (http://www.aspgd.org/). Introns are shown red and the direction of transcription is indicated by the arrow-like gene boxes. The inset represents a zoom in the boxed area, the *prnB–prnD* intergenic region, where the relative position of the two physiologically relevant PrnA- (green oval), CreA-, corresponding to *prn^d^*20 and *prn^d^*22 mutations, (purple rectangles) and AreA- binding sites (orange rectangles) are shown.

It is evident that the efficient utilization of external nutrients requires optimal coordination of the transcriptional and post translational regulation of the transport system. For efficient assimilation of a given nutrient, in addition to its uptake from the environment, the coordinated expression of the corresponding catabolic enzymes is required. A particularly well-studied case for this is the utilization of l-proline by *A. nidulans*. This fungus is able to utilize proline as both nitrogen and carbon source. Interestingly, as shown in Figure 3, the proteins involved in the uptake and the catabolism of l-proline map in five structural genes are tightly linked in a DNA region of about 13 kb in chromosome VII [68]. This *prn* clustering correlates with coordinated transcriptional regulation of these genes, potentially leading to the optimal coordination of the uptake of externally provided l-Pro and its enzymatic degradation into the cell. Proline is taken up through a specific transporter encoded by the *prnB* gene [11], paradigmatic member of the YAT family [15,17,21,29,51,57,69,70,71,72] and a lower-affinity and yet unidentified minor transport system [11,69]. Once inside the cell, proline is most likely to be transported to mitochondria through a proline uniporter and/or a proline/glutamate antiporter [73]. On the inner mitochondrial membrane, proline is converted in a flavin adenine dinucleotide-dependent manner to l-Δ^1^-pyrroline-5-carboxylate (P5C), a reaction catalyzed by a mitochondrial-resident l-proline oxidase encoded by the *prnD* gene. Subsequently, P5C is oxidized in a nicotinamide adenine dinucleotide-dependent manner to glutamate by a specific dehydrogenase encoded by the *prnC* gene. Moreover, another gene, *prnX,* has been detected by transcript analysis and sequencing in the *prn* cluster. *prnX* codes for a predicted 40 kDa protein that is not necessary for proline utilization and its function is hitherto unknown [68]. Finally, the *prnA* regulatory gene of the *prn* cluster (see below) encodes the pathway-specific positive acting regulatory factor, PrnA, which mediates specific induction by proline of *prn* structural genes [3,69,70,74,75].

A region of 1672 bp between the initiation codons of *prnB* [11] and *prnD* [76] genes (*prnB–prnC* intergenic region, DDBJ/EMBL/GenBank, Accession number U74465), is a bi-directional promoter of *prnB* and *prnD* genes, since mutations in PrnA-binding sites in this region have impact on transcription of both *prnD* and *prnB* ([68,70]). Moreover, as shown in Figure 3, *prnC* has the same direction of transcription as *prnB,* while *prnX* and *prnA* have the same direction of transcription as *prnD* [11,68,77]. 

The *prnB*, *prnC, prnX* and *prnD* are subject to pathway specific induction by proline, mediated by PrnA [70,74,75,77]. The expression *of prnB*, *prnD* and *prnC* and, to a lesser extent of *prnX*, is strictly dependent on the transcriptional activator of PrnA, and on proline induction relying on the efficient accumulation of proline by PrnB. In *A. nidulans* mycelia, transcription of *prnB* is highly induced by the low levels of proline, accumulated inside the cell by a minor transport system, only in the simultaneous absence of readily utilized nitrogen and carbon sources (see below). PrnA, the specific *trans*-transcriptional activator of the *prn* cluster, belongs to the class of Zn binuclear DNA binding regulators (Zn2Cys6). It differs from the isofunctional *S. cerevisiae* protein Put3p, both in its unique binding specificity and in the requirement of induction for in vivo DNA binding [75,78]. It binds as the dimer to CCGG-N-CCGG inverted repeats, and to CCGG-6/7N-CCGG direct repeats in three sites of the *prnD–prnB* intergenic region, and three in the *prnB–prnC* intergenic region. Recognition of the direct repeats necessitates the PrnA dimerization and linker elements, while the recognition of the CCGG-N-CCGG inverted repeats depends crucially on the PrnA Zn binuclear cluster and/or residues within the amino-terminal tail of PrnA [78]. In the absence of the proline-inducer, the PrnA protein—expressed in low levels by its weak promoter—is localized in the nucleus [79] and the induction by proline specifically elicits its DNA-binding [78].

The *prnB* gene is also subject to two wide domain controls, nitrogen metabolite repression (NMR) [80,81,82,83] and carbon catabolite repression (CCR) [80,84,85]. Nitrogen metabolite repression is mediated by the product of *areA* gene, and carbon catabolite repression by the product of the *creA* gene. 

The *areA* gene of *A. nidulans* encodes a GATA family transcriptional activator (AreA) ([80,81,86,87,88,89,90] and references therein) required for the expression of more than 100 genes coding for the enzyme and permeases involved in catabolic pathways of less favored nitrogen metabolites in the absence of a readily utilized “rich” or repressing nitrogen source (ammonium or l-glutamine). AreA and its homologous GATA transcription factors bind to HGATAR consensus binding sites [91] upstream of the cognate regulated genes. AreA DNA binding and transcriptional activation are affected by repressing nitrogen sources at multiple levels, including transcriptional, post-transcriptional and post-translational [88,92,93,94]. AreA is essential for full transcriptional activation of genes involved in the utilization of metabolites that can serve as sole nitrogen sources, irrespective of the carbon source utilized. On the contrary, for most genes involved in the utilization of metabolites that can serve as both nitrogen and carbon sources, AreA is only necessary for conditions of carbon catabolite repression [76,80,95,96]. GATA factors include AreA [91] and its *Neurospora crassa* Nit2 proteins and *S. cerevisiae* homologues [83,97,98].

On the other hand, CreA, a two zinc finger of the C2H2 class protein [84,99], is a general transcriptional repressor of genes involved in carbon catabolism of secondary or less favored carbon sources in the presence of a “preferable” or repressing carbon source (glucose or fructose). The two zing fingers of CreA are very similar to those present in the transcriptional regulator MIG1 of *S. cerevisiae*, which, however, is involved in the carbon catabolite repression of only some *S. cerevisiae* genes (e.g., GAL4 and SUC) [100,101], and to a lesser extent to the mammalian Egrl and Egr2 proteins and to the Wilms’ tumor protein [99].

In general, expression of genes involved in the uptake and utilization of less favored carbon sources is repressed by glucose, irrespective of the nitrogen source present in the medium or the *areA^−^* allele present in the strain [85]. This is exemplified by the ethanol utilization regulon *alc* of *A. nidulans*, where transcriptional activation of clustered genes is mediated by the AlcR pathway specific activator in the presence of the physiological inducer acetaldehyde, while transcriptional repression is mediated directly, via the binding of the repressor CreA to its cognate targets located in the *alc* gene promoters, and indirectly, by down-regulating the pathway specific regulatory *alcR* gene [102,103].

Conversely, expression of genes involved in the utilization of compounds which only serve as nitrogen sources is repressed by ammonium or glutamine irrespectively of the carbon source or the presence of a *creA^d^* (carbon catabolite-derepressed) mutation [80,84], as exemplified by the transcriptional activation of nitrate assimilating genes (*niiA*, *niaD*). Expression of the latter genes depends on the cooperative action of AreA and the pathway-specific activator NirA. Using this system, it was shown that the intracellular concentration of glutamine (Gln) inversely correlates with the known AreA activities [104].

Since proline can serve as both carbon and nitrogen source in *A. nidulans*, *prnB* expression is subject to both NMR and CCR. More precisely, *prnB* expression is fully repressed only when both repressing nitrogen (ammonium) and carbon sources (glucose) are simultaneously present in the growth medium. In other words, the AreA activator is only required for the expression of *prnB* in the presence of an active CreA repressor bound to its cognate binding sites. It has been proposed that this is achieved through a mechanism where CreA prevents the activity of a positively acting ADA (absolute dependence on AreA) element, which operates besides AreA in the *prnD–prnB* intergenic region. An alternative explanation is that ADA is a DNA sequence that maintains the promoter region in a transcriptionally competent “open” configuration, and is ‘closed’ at a distance by CreA, while binding of AreA to sites near the transcription start points of *prnB* and *prnD* can by-pass this effect [76]. Although the synthesis of *prnC* and *prnD* products is also repressed, the limiting step for proline utilization under repressing conditions is proline uptake, since *prn^d^* mutations (derepressed), mapping in the *prnD–prnB* intergenic region result in derepression of *prnB,* and also of *prnD* and *prnC* [105,106]. Simultaneous repression by ammonium and glucose does not affect the in vivo binding of PrnA in the promoters of *prn* structural genes [78] and derepression of *prnD* and/or *prnC*, is the result of reversal of the inducer exclusion, acting via the derepression of *prnB* [105,107]. Moreover, the partial nucleosomal positioning observed under double repressing conditions depends on the CreA repressor’s binding to two specific *cis*-acting sites [108] (see below).

The *prnD–prnB* intergenic region contains seven CreA binding sites, with the binding consensus 5’-SYGGRG-3’, two of which have been shown to be necessary for the repression of *prn* cluster in vivo [109] (Figure 3). One of them is defined by the operator derepressed mutations *prn^d^* 20, 22. These transition mutations result in derepression of *prnB* at the level of mRNA accumulation and affect directly or indirectly the levels of *prnC* and *prnD* [105,106,110]. Interestingly, they are the first eukaryotic homologue of operator/derepressed mutations to be described [111]. The second one is a divergently oriented sequence, separated by one base pair from the sequence defined by *prn^d^* mutations [109]. Mutations on either of the two relevant CreA binding sites of the *prnD–prnB* intergenic region, suppresses an *areA* null mutation for utilization of proline as the sole nitrogen source in the presence of glucose [76,105,106,109,110].

Additionally, the overall *prnD–prnB* intergenic region contains 17 GATA sites of which the two *prnB* proximal sites are involved in setting the maximal level of *prnB* transcription and bypassing of CreA repression [112]. Moreover, the *prnB* proximal TATA box acting only on *prnB*, is not essential for transcription, while neither AreA nor PrnA activity is restricted to the recruitment of the TATA binding protein to the TATA box [112].

Overall, the above genetic data but also those from gel shifts, in vitro and in vivo foot-printing, as well as interference assays are consistent with a model where pathway-specific induction mediated by the PrnA activator would act on each gene of the *prn* cluster, while the general factors CreA and AreA act primarily, and perhaps exclusively, by regulating the expression of the proline transporter gene, *prnB* [105,107]. However, genetic evidence [111] suggest that AreA and CreA, “acting at a distance”, affect transcription of *prnC*, while *prnX* is under PrnA regulation ([68,77] and S. Demais, I. Garcia, V. Gavrias, D. Gómez, N., Oestreicher and C. Scazzocchio, unpublished).

Besides the well-established mechanism of proline induction mediated by the *prnA* gene [3,74,110], the *prnB* expression is regulated by three other mechanisms [70]. 

One mechanism is *prnA*-partially independent and is present in germinating conidia of *A. nidulans*. It has been found that *prnB* expression is transiently activated early during germination via a system(s) that does not affect the expression of other genes involved in proline catabolism. This *prnB* activation is correlated with an increase in the steady state of *prnB* mRNA, implying that the new system(s) may be affecting *prnB* transcription and/or *prnB* mRNA stability [69,70].

The second mechanism of *prnB* transcriptional induction is *prnA*-independent and amino-acid starvation dependent. Mutational evidence strongly suggests the involvement of the general regulator CpcA/Gcn4 in the activation of this mechanism, as it is lost in mutants where the only canonical CpcA [113,114,115,116] putative binding site present in the *prnD–prnB* intergenic region is mutated. The CpcA protein of *A. nidulans* is a member of the c-Jun-like transcriptional activator family and is functionally exchangeable for the general amino acid control transcriptional activator Gcn4p of *S. cerevisiae.* Moreover, previous studies have shown that chromatin rearrangements in the *prnD–prnB* intergenic region depend on the transcription factors. More precisely, in the *prnD*–*prnB* intergenic region, eight nucleosomes lost their positioning upon proline induction while simultaneous carbon and nitrogen metabolite repression resulted in partial nucleosome repositioning. Chromatin restructuring is strictly dependent on the PrnA but not on AreA, which was shown to be essential for chromatin opening in the *A. nidulans* nitrate regulon [89,117]. Nucleosome positioning is completely lost when CreA is nonfunctional and partially lost in the presence of a specific inhibitor of histone H3 deacetylase [118]. However, nucleosome positioning and histone H3 acetylation are independent processes in the *prnD–prnB* bidirectional promoter [118]. Under double repressing conditions, partial nucleosomal positioning is observed. This depends on the CreA repressor’s binding to two specific *cis*-acting sites [118]. The above results show that amino acid starvation is at least partially responsible for the *prnA*-independent accumulation of the *prnB* message through a CpcA/Gcn4-dependent activation mechanism, establishing that the expression of a transporter gene responds to regulatory proteins involved in both catabolic (PrnA) and biosynthetic (CpcA/Gcn4) pathways [70]. The third mechanism is responsive to the amino acid, or at least to proline, starvation and is *prnA*-dependent [72]. 

## Figures and Tables

**Table 1:** Yeast amino acid transporters (YATs) that have been studied by structural modelling.

Name	Organism	Specificity	Reference
Bap2	*S. cerevisiae*	Branched chain and aliphatic amino acids	[42]
Can1	*S. cerevisiae*	High affinity for Arg, low affinity for Lys and His	[16,40,44]
CgCyn1	*C. glabrata*	Cystine	[43]
Gap1	*S. cerevisiae*	General amino acid permease	[40]
Lyp1	*S. cerevisiae*	Lys	[16]
PrnB	*A. nidulans*	Pro	[17,29]
Put4	*S. cerevisiae*	High affinity for Pro, also transports Ala, Gly, GABA and L-AZC	[17]
Tat2	*S. cerevisiae*	High affinity for Trp and Tyr, also transports Phe, Ala, Gly and Cys	[41]
